# Priming of Cardiopulmonary Bypass with Human Albumin Decreases Endothelial Dysfunction after Pulmonary Ischemia–Reperfusion in an Animal Model

**DOI:** 10.3390/ijms23168938

**Published:** 2022-08-11

**Authors:** Jean Selim, Mouad Hamzaoui, Antoine Ghemired, Zoubir Djerada, Laurence Chevalier, Nicolas Piton, Emmanuel Besnier, Thomas Clavier, Anaïs Dumesnil, Sylvanie Renet, Paul Mulder, Fabien Doguet, Fabienne Tamion, Benoît Veber, Jérémy Bellien, Vincent Richard, Jean-Marc Baste

**Affiliations:** 1 Normandie University, UNIROUEN, INSERM U1096, 76000 Rouen, France; 2 Department of Anesthesiology and Critical Care, Rouen University Hospital, 76000 Rouen, France; 3 Normandie University, UNIROUEN, CNRS, GPM-UMR 6634, 76000 Rouen, France; 4 Department of Pathology, Rouen University Hospital, 76000 Rouen, France; 5 Department of Medical Intensive Care, Rouen University Hospital, 76000 Rouen, France; 6 Department of Thoracic Surgery, Rouen University Hospital, 76000 Rouen, France

**Keywords:** lung transplantation, ischemia–reperfusion, cardiopulmonary bypass, endothelial dysfunction, glycocalyx, human albumin, hypertonic sodium lactate

## Abstract

The routine use of mechanical circulatory support during lung transplantation (LTx) is still controversial. The use of prophylactic human albumin (HA) or hypertonic sodium lactate (HSL) prime in mechanical circulatory support during LTx could prevent ischemia–reperfusion (IR) injuries and pulmonary endothelial dysfunction and thus prevent the development of pulmonary graft dysfunction. The objective was to investigate the impact of cardiopulmonary bypass (CPB) priming with HA and HSL compared to a CPB prime with Gelofusine (GF) on pulmonary endothelial dysfunction in a lung IR rat model. Rats were assigned to four groups: IR-CPB-GF group, IR-CPB-HA group, IR-CPB-HSL group and a sham group. The study of pulmonary vascular reactivity by wire myograph was the primary outcome. Glycocalyx degradation (syndecan-1 and heparan) was also assessed by ELISA and electron microscopy, systemic and pulmonary inflammation by ELISA (IL-1β, IL-10, and TNF-α) and immunohistochemistry. Clinical parameters were evaluated. We employed a CPB model with three different primings, permitting femoral–femoral assistance with left pulmonary hilum ischemia for IR. Pulmonary endothelium-dependent relaxation to acetylcholine was significantly decreased in the IR-CPB-GF group (11.9 ± 6.2%) compared to the IR-CPB-HA group (52.8 ± 5.2%, *p* < 0.0001), the IR-CPB-HSL group (57.7 ± 6.3%, *p* < 0.0001) and the sham group (80.8 ± 6.5%, *p* < 0.0001). We did not observe any difference between the groups concerning glycocalyx degradation, and systemic or tissular inflammation. The IR-CPB-HSL group needed more vascular filling and developed significantly more pulmonary edema than the IR-CPB-GF group and the IR-CPB-HA group. Using HA as a prime in CPB during Ltx could decrease pulmonary endothelial dysfunction’s IR-mediated effects. No effects of HA were found on inflammation.

## 1. Introduction

Lung transplantation (LTx) is the reference treatment for patients with terminal respiratory failure. Primary graft dysfunction (PGD), which usually occurs within 72 h after lung transplantation, is one of the main complications of transplantation with high morbidity and mortality. The etiologies of PGD are multiple, but pulmonary ischemia–reperfusion (IR) and endothelial dysfunction are the main causes [1,2,3]. Analysis of the literature shows conflicting data on the routine use of mechanical circulatory support in lung transplantation. This topic is still controversial in LTx teams [4]. In a cardiopulmonary bypass (CPB) model associated with pulmonary IR in rats, we have recently shown that CPB significantly increased the effects of pulmonary IR on pulmonary vascular dysfunction, on systemic and tissular inflammation, and on glycocalyx degradation [5]. Indeed, the study of pulmonary arteries’ vasoreactivity has shown that pulmonary endothelium-dependent relaxation to acetylcholine was markedly reduced in the group with CPB associated with pulmonary IR compared to the IR group, the CPB or the sham group. This relaxation pathway was mainly mediated by nitric oxide (NO). CPB associated with pulmonary IR was also involved in an increase systemic inflammation (IL-1β and IL-10), in tissular lung inflammation (macrophage infiltration) and in glycocalyx degradation (syndecan-1) compared to the pulmonary IR group. In this first study, the CPB priming solution was realized with 4% Gelofusine.

Many strategies to prevent CPB-related systemic inflammatory response or endothelial dysfunction have been developed [6]. One of them is the choice of the fluid used for the priming of the CPB circuit. Thus, human albumin (HA) and hypertonic sodium lactate (HSL) priming could be interesting in our model. In addition to these oncotic properties, HA may have a protective effect on the endothelium and the glycocalyx degradation or systemic inflammation during sepsis [7,8]. During cardiac surgery, the CPB prime with HA could decrease endothelial dysfunction and the systemic inflammatory process [9].

Fluids containing lactate may also be of interest. Indeed, several data support that lactate is a major metabolite during inflammation and represents an alternate source of energy for various organs, including the heart or the lungs. Certain experimental and human studies have reported the positive effects of molar hypertonic sodium lactate (HSL) in different situations, such as brain injury or cardiac dysfunction, with a significant beneficial effect on endothelial dysfunction and microcirculation [10,11]. However, HSL has never been studied in a CPB experimental model or as a prime in cardiac surgery to our knowledge.

In this context, the use of prophylactic HA or HSL prime in mechanical circulatory support during LTx could prevent IR injuries, pulmonary endothelial dysfunction, systemic inflammatory response and thus prevent the development of PGD. The primary objective of this study was to investigate the impact of the CPB priming with HA and HSL compared to a CPB prime with Gelofusine solution on pulmonary endothelial dysfunction in a model of pulmonary IR in rats. Secondary objectives were to study the glycocalyx degradation and systemic and tissue inflammation.

## 2. Results

### 2.1. Effect of Priming Solution on Pulmonary Endothelial Function

**Figure 1:**
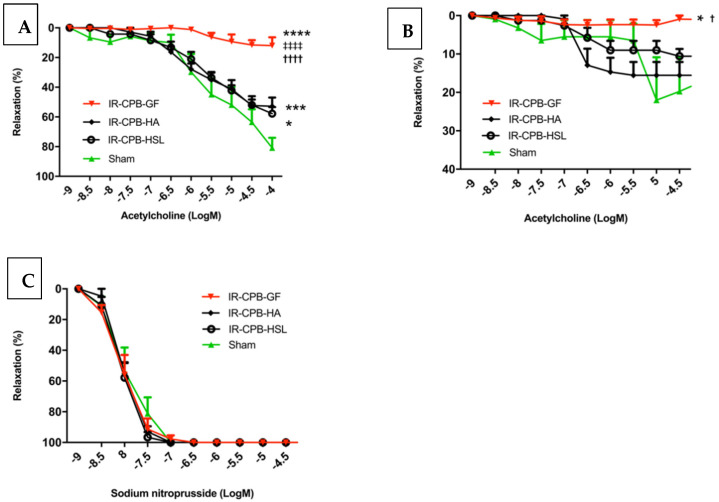
In vitro vascular responses. (**A**) Relaxation to acetylcholine after precontraction with phenylephrine (endothelium-dependent relaxation). * *p* < 0.05 vs. sham. *** *p* < 0.001 vs. sham. **** *p* < 0.0001 vs. sham. ^††††^ *p* < 0.0001 vs. HA. ^‡‡‡‡^ *p* < 0.0001 vs. HSL. (**B**) Relaxation to sodium nitroprusside after precontraction with phenylephrine (endothelium-independent relaxation). (**C**) Relaxation to acetylcholine after precontraction with phenylephrine with L-NNA. * *p* < 0.05 vs. sham. ^†^ *p* < 0.05 vs. HA. For statistical analyses, ANOVA was used for repeated measurements, followed by Tukey’s multiple comparison (post hoc) testing. The concentrations of acetylcholine and sodium nitroprusside were expressed by the logarithmic decimal value of their molarity (**A**–**C**). A result with *p* < 0.05 was considered to be statistically significant. For (**A**–**C**), responses are expressed as percentage relaxation of phenylephrine-induced precontraction (mean ± SEM) (*n* = 5 per group). Abbreviations: CPB, cardiopulmonary bypass; IR, left lung ischemia–reperfusion; HA, human albumin; HSL, hypertonic sodium lactate; Log [M], logarithm decimal of molarities.

Pulmonary endothelium-dependent relaxation to acetylcholine was significantly decreased in the IR-CPB-GF group (11.9 ± 6.2%) compared to the IR-CPB-HA group (52.8 ± 5.2%, *p* < 0.0001) (Figure 1A). Pulmonary endothelium-dependent relaxation to acetylcholine was significantly decreased in the IR-CPB-GF group (11.9 ± 6.2%) compared to the IR-CPB-HSL group (57.7 ± 6.3%, *p* < 0.0001) (Figure 1A). There was no difference between the IR-CPB-HA and IR-CPB-HSL group. Investigation of pulmonary endothelium-independent relaxation to sodium nitroprusside (SNP) indicated no difference between groups, suggesting no alteration of smooth muscle cells (Figure 1B).

### 2.2. Role of NO

To explore the pathways implicated in endothelium-dependent relaxation in our work, we incubated pulmonary arteries with L-NG-nitro-arginine (L-NNA) to limit NO production by inhibiting NO synthase. We observed that endothelium-dependent relaxation of pulmonary arteries in the sham group decreased from 80.8 ± 6.5% to 17.1 ± 4.4% (*p* < 0.01). In the IR-CPB-GF group, vasorelaxation was completely abolished during NO synthase inhibition. The diminution of the endothelium-dependent relaxation was significantly less important in the sham group (17.1 ± 4.4%) and the IR-CPB-HA group (15.6 ± 3.8%) compared to the IR-CPB-GF group (1.0 ± 4.4%, *p* < 0.05). There was no significant difference between the IR-CPB-GF and the IR-CPB-HSL group (Figure 1C).

### 2.3. Glycocalyx Degradation

**Figure 2:**
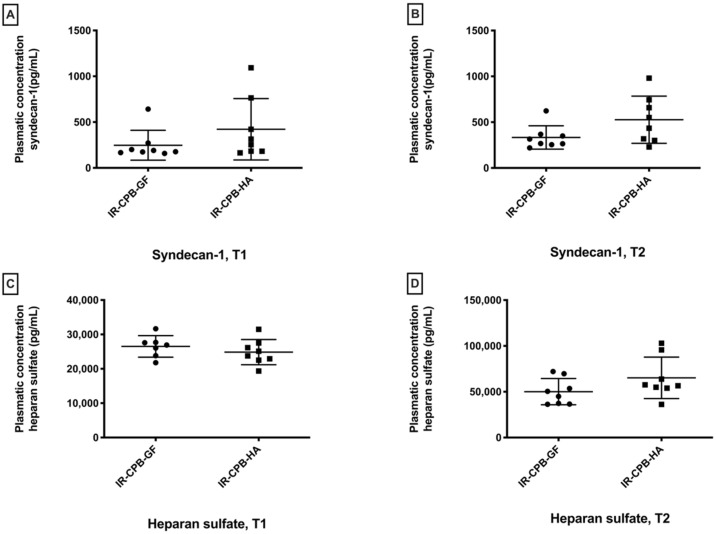
Plasmatic glycocalyx degradation. (**A**) Plasma concentration of syndecan-1 at T1. (**B**) Plasma concentration of syndecan-1 at T2. (**C**) Plasma concentration of heparan sulfate at T1. (**D**) Plasma concentration of heparan sulfate at T2. For statistical analyses, ANOVA was used, followed by Tukey’s multiple comparison (post hoc) testing. A result with *p* < 0.05 was considered to be statistically significant. Data are presented as mean ± SD. Abbreviations: CPB, cardiopulmonary bypass; IR, left lung ischemia–reperfusion; HA, human albumin.

For syndecan-1, we did not observe any significant differences between the IR-CPB-GF group and the IR-CPB-HA group at T1 and T2 (T2, IR-CPB-GF: 332.7 ± 127.5 pg/mL; IR-CPB-HA: 526.8 ± 257.3 pg/mL, *p* = 0.10) (Figure 2A,B). For heparan sulfate, we did not observe any significant differences between the IR-CPB-GF group and the IR-CPB-HA group at T1 and T2 (T2, IR-CPB-GF: 47839 ± 11541 pg/mL, IR-CPB-HA: 65196 ± 22578 pg/mL, *p* = 0.16) (Figure 2C,D). Transmission electron microscopy (TEM) glycocalyx evaluations are presented in Supplementary Material S1 and Supplementary Figures S1 and S2.

### 2.4. Inflammatory Response

**Figure 3:**
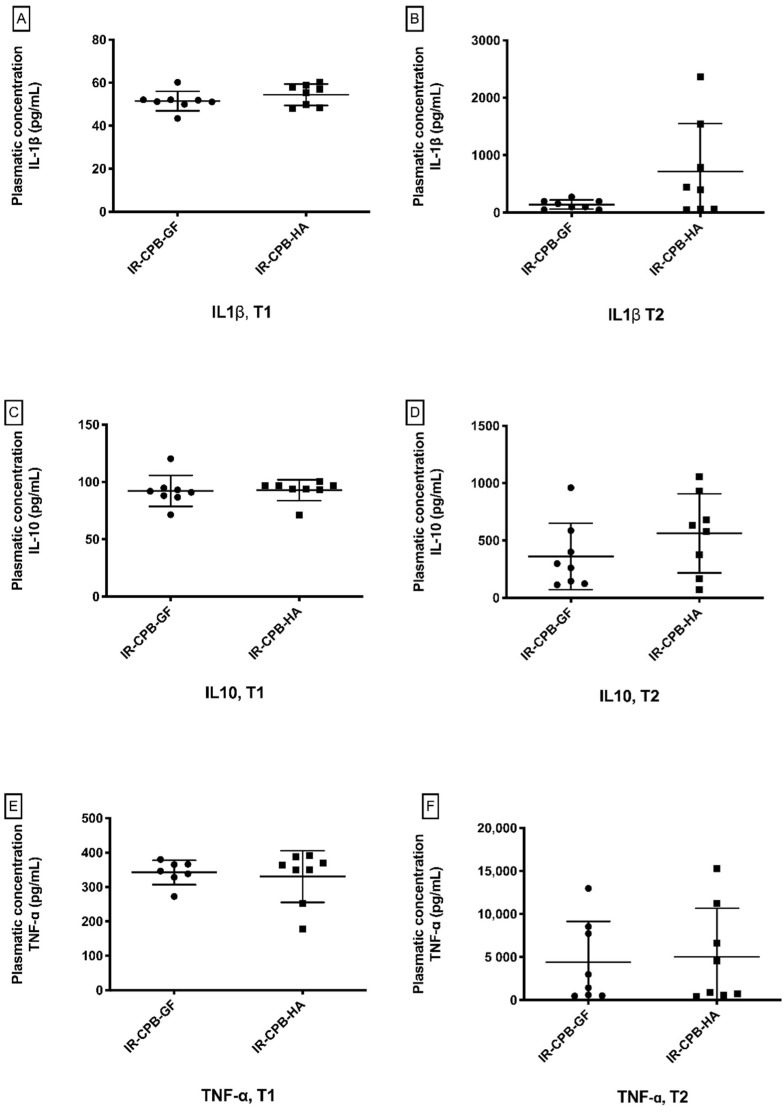
Systemic inflammation. (**A**) Mean value of IL-1β plasma level at T1. (**B**) Mean value of IL-1β plasma level at T2. (**C**) Mean value of IL-10 plasma level at T1. (**D**) Mean value of IL-10 plasma level at T2. (**E**) Mean value of TNF-α plasma level at T1. (**F**) Mean value of TNF-α plasma level at T2. For statistical analyses, ANOVA was used, followed by Tukey’s multiple comparison (post hoc) testing. A result with *p* < 0.05 was considered to be statistically significant. Data are presented as mean ± SD. Abbreviations: CPB, cardiopulmonary bypass; IR, left lung ischemia–reperfusion; HA, human albumin.

We did not find a significant difference between the IR-CPB-GF group and the IR-CPB-HA group at T1 for TNF-α, IL-1β, and IL-10. TNF-α levels (IR-CPB-GF: 4408 ± 4741 pg/mL, IR-CPB-HA: 5025 ± 5645 pg/mL, *p* = 0.95), IL-1β levels (IR-CPB-GF: 140.8 ± 79.6 pg/mL, IR-CPB-HA: 715.9 ± 836 pg/mL, *p* = 0.16) and IL-10 levels (IR-CPB-GF: 361.8 ± 290.2 pg/mL, IR-CPB-HA: 562.7 ± 345 pg/mL, *p* = 0.27) were not significantly different between the GF and the HA groups at T2 (Figure 3).

### 2.5. Histology

**Figure 4:**
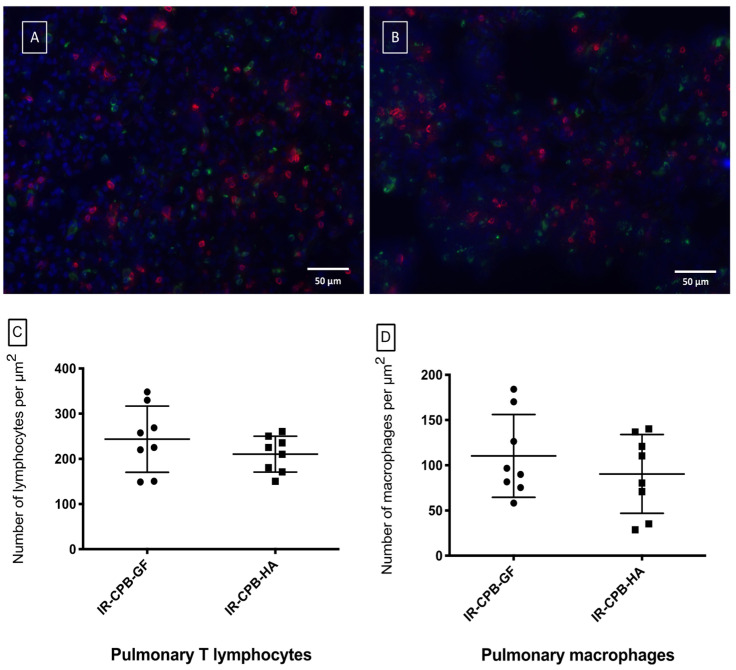
Immunohistology (magnification x40, scale bar = 50 μm). Fluorescent immuno-histological marking of the pulmonary parenchyma of the IR-CPB-GF group (**A**) and the IR-CPB-HA group (**B**). For all the groups, macrophages (CD68) are marked in green and lymphocytes (CD3) are marked in red. (**C**) Pulmonary T lymphocytes (CD3) of the left lung tissue. (**D**) Pulmonary macrophages (CD68) of the left lung tissue. For statistical analyses, ANOVA was used, followed by Tukey’s multiple comparison (post hoc) testing. A result with *p* < 0.05 was considered to be statistically significant. Data are presented as mean ± SD. Abbreviations: CPB, cardiopulmonary bypass; IR, left lung ischemia–reperfusion; HA, human albumin.

Examination of the lung sections indicated that there was no difference between the IR-CPB-GF (110 ± 45.77 macrophages/μm^2^) and IR-CPB-HA (90.47 ± 43.59 macrophages/μm^2^, *p* = 0.50) groups for macrophages infiltration and for T lymphocytes infiltration (IR-CPB-GF: 243.60 ± 73.38; IR-CPB-HA: 210.50 ± 39.64 lymphocytes/μm^2^, *p* = 0.50) (Figure 4C,D).

### 2.6. Clinical Parameters

**Figure 5:**
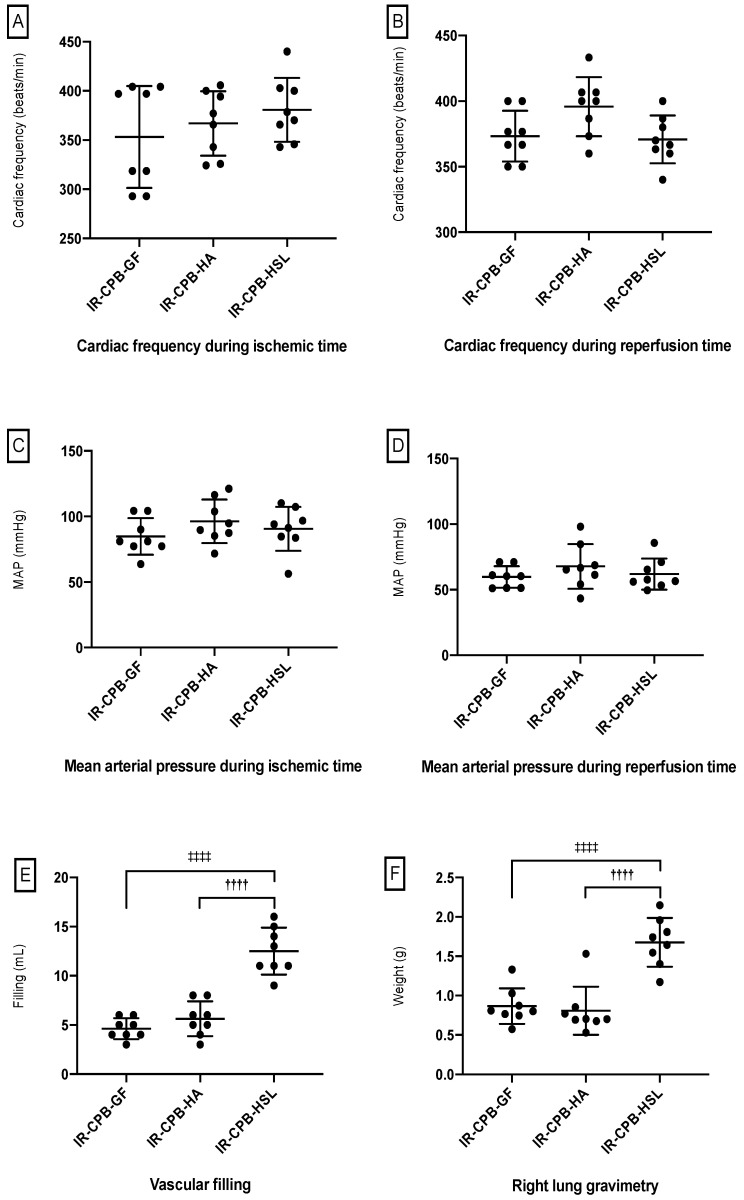
Clinical parameters. (**A**) Heart rate during ischemia. (**B**) Heart rate during reperfusion. (**C**) Mean arterial pressure during ischemia. (**D**) Mean arterial pressure during reperfusion. (**E**) Vascular filling. ^††††^ *p* < 0.0001 vs. HA. ^‡‡‡‡^ *p* < 0.0001 vs. HSL (**F**) Right lung gravimetry. ^††††^ *p* < 0.0001 vs. HA. ^‡‡‡‡^ *p* < 0.0001 vs. HSL. For statistical analyses, ANOVA was used, followed by Tukey’s multiple comparison (post hoc) testing. A result with *p* < 0.05 was considered to be statistically significant. Data are presented as mean ± SD. Abbreviations: CPB, cardiopulmonary bypass; IR, left lung ischemia–reperfusion; HA, human albumin; HSL, hypertonic sodium lactate.

We did not find a significant difference between the IR-CPB-GF, the IR-CPB-HA and the IR-CPB-HSL during the ischemic time and the perfusion time for mean arterial pressure (MAP) and the heart rate (Figure 5A–D). We observed significantly higher needs for fluid infusion to reach MAP objectives in the IR-CPB-HSL group (12.50 ± 2.40 mL) compared to the IR-CPB-GF (4.62 ± 1.06 mL, *p* < 0.0001) group and the IR-CPB-HA group (5.62 ± 1.76 mL, *p* < 0.0001) (Figure 5E). A higher pulmonary wet/dry ratio was observed in the IR-CPB-HSL group (1.67 ± 0.31) compared to the IR-CPB-GF (0.86 ± 0.22, *p* < 0.0001) group and the IR-CPB-HA group (0.80 ± 0.30, *p* < 0.0001) (Figure 5F).

## 3. Discussion

The major finding of the study is that HA used as a priming agent in CPB instead of Gelofusine decreases pulmonary endothelial dysfunction in the pulmonary artery in our model simulating pulmonary IR during LTx. When mechanical circulatory support is required for LTx, the question of the choice of the priming may be considered.

### 3.1. Fluid Therapy by Human Albumin

Regarding the analysis of pulmonary vascular function, we found that the IR-CPB-GF group was associated with severe endothelial dysfunction revealed by a reduced vasorelaxation to acetylcholine (11.9 ± 6.2%). This endothelial dysfunction was significantly lower in the IR-CPB-HA group (52.8 ± 5.2%). The NO pathway was mainly involved in the vasorelaxation of pulmonary arteries. Indeed, we found the fundamental role of NO in the sham group with a maximum relaxation close to 80%, suggesting that the relaxation of the pulmonary arteries is mainly mediated by NO and those other pathways are involved in 20% of the relaxations. Relaxation was completely abolished by NO synthase in the IR-CPB-GF group (1.0 ± 4.4%) and not completely abolished in the sham group (17.1 ± 4.4%) and in the IR-CPB-HA group (15.6 ± 3.8%), suggesting that alternative pathways are preserved with albumin fluid therapy. These results are consistent with other studies that found a protective effect of albumin on vasorelaxation of the mesenteric arteries [7,8,12]. Our results suggest that HA may have a protective effect on the pulmonary endothelium by preserving endothelial vasorelaxation. Several preclinical studies have shown that HA has a role in endothelial protection and have explored its mechanisms of action in ischemia–reperfusion, sepsis, hemorrhagic shock, and mechanical circulatory support [13,14,15]. In addition, various in vitro and in vivo studies have shown the multifactorial properties of albumin on the integrity of the glycocalyx, vascular permeability and anti-inflammatory effects [16,17]. Albumin is physiologically bound with the glycocalyx, protecting it from shedding and contributing to its maintenance. In our study, we did not observe any significant difference concerning the biomarkers of glycocalyx degradation (syndecan-1 and heparan sulfate) between the Gelofusine group and the HA group. Several hypotheses may explain this result. First, the aggressiveness of our experiment did not allow long IR times (30 min of ischemia and 15 min of reperfusion) and was not sufficient to demonstrate a difference between the groups in plasma glycocalyx degradation. Second, other markers, such as cell junction markers (ICAM and VCAM), would have been relevant to study vascular permeability and would have been expressed early in this model. Finally, it is important to highlight that HA fluid therapy is not associated with greater degradation of the glycocalyx than Gelofusine, which is currently the most widely used colloid in cardiopulmonary bypass. Although high-quality randomized studies are still lacking to clarify the indications for albumin in cardiac surgery with CPB, the existing studies demonstrated its efficacy and safety of use [9,18]. Pesonen et al. did not find a difference for major adverse events over the following 90 days in a recent randomized monocentric study comparing 4% albumin and ringer acetate as a CPB prime. Even if no difference was found concerning the incidence of clinically important adverse cardiac events, albumin significantly reduced the postoperative plasma creatine kinase-MB. This finding could be explained by the protective role of albumin on the coronary glycocalyx [19]. Even if, to our knowledge, no study using HA has been used on mechanical circulatory support during lung transplantation, it is important to highlight that human albumin is one of the main components of Steen’s solution used in the ex vivo lung perfusion with the Toronto protocol [20].

Regarding systemic inflammation, we did not find any significant difference between the two groups concerning TNF-α, IL-1β and IL-10. Considering the endothelial dysfunction induced by this model, this result may appear surprising, indeed, endothelial dysfunction is also associated with the activation of endothelial cells that become pro-inflammatory, increasing the expression of adhesion molecules, producing monocyte chemoattractant protein-1 (MCP-1), increasing leukocyte transmigration and activation, and involving cytokines [21]. Our findings can be explained by the short IR time of the lung but also by the HA concentration we used (HA 5%). Indeed, some authors describe a protective effect of albumin against systemic inflammation with higher albumin concentrations (HA 20%) [8].

### 3.2. Fluid Therapy by Hypertonic Sodium Lactate

Concerning the study of vasorelaxation to acetylcholine of the pulmonary arteries, we found that the HSL group was significantly associated with less severe endothelial dysfunction compared to the Gelofusine group. However, there was no difference found with HA and the main pathway involved was also the NO pathway. These results are encouraging for the protective effect of HSL on the endothelium. However, we did not continue the investigations with the exploration of glycocalyx degradation and systemic inflammation (biomarkers and TEM). Indeed, analysis of the clinical and hemodynamic parameters of the HSL group did not clinically convince us. The HSL group required significantly more filling than the other groups to obtain the correct perfusion pressure. The clinical consequence was significantly greater pulmonary edema in this group. These results can be explained by the oncotic effects of HSL, which is hyperosmolar and leads to a significant passage of water from the vascular sector to the interstitial sector [22,23]. These clinical observations, therefore, are opposed to what is expected in lung transplantation, and it did not appear relevant to continue the analysis by molecular biology, immunohistochemistry, or TEM in this group. The use of undiluted HSL in the CPB probably explains the oncotic problems we encountered. A dilution would have been considered to benefit from the protective effects on the endothelium and to limit the formation of pulmonary edema.

### 3.3. Limits

Our work has a number of limitations. First, this model corresponds to warm ischemia and concerns only one lung. Second, the aggressiveness of the model did not allow us to extend the duration of the experiment. Third, we could not explore other pathways than the NO pathway. Fourth, our TEM analysis of the glycocalyx is only qualitative and not quantitative. Fifth, we did not explore glycocalyx degradation by molecular biology for HSL. Further studies with HSL diluted in CPB would be interesting to conduct. Finally, we did not explore the degradation of elastin and collagen in the vessel walls which is an important part of endothelial dysfunction. Indeed, inflammatory cells produce metalloproteinases that regulate cardiovascular remodeling enabling the degradation of elastin and collagen in the vessel wall [21].

## 4. Materials and Methods

### 4.1. Animal Care and Study Groups

**Figure 6:**
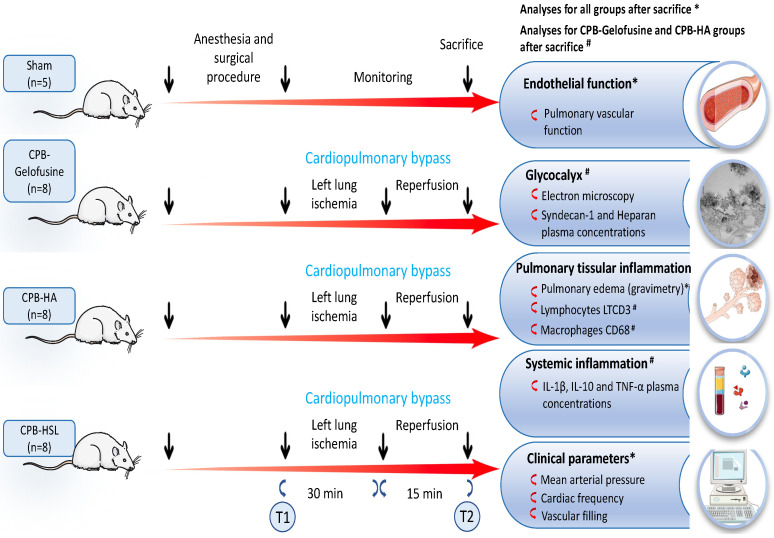
Schematic diagram of the experimental procedure and sampling points. T1, Time 1 corresponding to left lung IR with CPB and samples obtained at the end of anesthesia-surgical procedure. T2, Time 2 corresponding to samples obtained at the end of the experiment. Abbreviations: CPB, cardiopulmonary bypass; IR, left lung ischemia–reperfusion; HA, human albumin; HSL, hypertonic sodium lactate. * Analyses for all groups after sacrifice. ^#^ Analyses for CPB-Gelofusine and CPB-HA groups after sacrifice.

Male Wistar rats (Laboratoires Janvier, Saint Berthevin, France) weighing 380 to 450 g were employed. They received humane care in accordance with the Guide for the Care and Use of Laboratory Animals published by the US National Institutes of Health (NIH Publication No. 85–23, revised 1996), and the study was approved by the French Ministry of Higher Education and Research-Directorate General for Research and Innovation (No. APAFIS 2016102718162386-V2). All procedures were performed in accordance with the French ethics committee as well as with the guidelines of the European Parliament Directive No. 2010/63/EU and the Council for the protection of animals used for scientific purposes, under the supervision of an authorized investigator. All animals had free access to standard potable tap water. Animals were maintained at a constant temperature of 21 °C with a 14/10 h dark/light cycle. Rats were divided into four groups and each group had an association of left pulmonary IR and CPB with a different prime solution: Gelofusine prime (IR-CPB-GF, *n* = 8), HA prime (IR-CPB-HA group, *n* = 8) and HSL prime (IR-CPB-HSL group, *n* = 8). A sham group was also constituted (*n* = 5). A schematic representation of the study design is shown in Figure 6.

### 4.2. Surgical Procedure

The surgical procedure was performed as previously described and available in Supplementary Material S2 [5,24]. Rats were anesthetized with an intraperitoneal injection of xylazine (10 mg/kg) and ketamine (80 mg/kg). The left carotid was cannulated with a 22-gauge catheter for continuous monitoring of heart rate, blood pressure and drug delivery. For mechanical ventilation, a tracheotomy was performed. The right femoral artery and the right femoral vein were cannulated respectively by a 22-gauge catheter and a 16-gauge catheter. For CPB, we used a CPB sterile circuit including a roller pump, a cardiotomy reservoir consisting of a 5 mL syringe, an oxygenator and tubing lines. The circuit was aseptically set up and free from air bubbles with a 10 mL solution of either 4% Gelofusine solution (4% Gelofusine^®^, B. Braun, Melsungen, Germany), 5% HA (5% Human Albumin, Vialebex^®^, LFB, Paris, France) or 11.2% HSL (11.2% sodium lactate (1000 mmol/L of sodium + 1000 mmol/L of lactate, APHP) according to the group. The vascular filling was realized with Gelofusine, HA, or HSL depending of the group.

A left thoracotomy was then performed in each group. The left pulmonary hilum was carefully tightened with a lasso for ischemia and released for reperfusion. Rats were exposed to 30 min of left pulmonary ischemia and 15 min of reperfusion. The rats were euthanized at the end of the study, for the sham group, rats were euthanized 45 min after conditioning. Blood samples were collected in all groups at two time points: (T1) immediately after the surgical procedure and before CPB, (T2) before the sacrifice.

### 4.3. Pulmonary Vascular Studies

Pulmonary vascular function was evaluated as previously described [5,25]. A 2–3 mm segment of second-order intralobar pulmonary artery (internal diameter < 700 μm), was meticulously dissected and mounted on a myograph (DMT^®^, Aarhus, Denmark). After normalization, responses to acetylcholine (10^−9^ to 3 × 10^−5^ mol/L) and to sodium nitroprusside (SNP) (10^−9^ to 3 × 10^−5^ mol/L) were acquired in segments pre-contracted with phenylephrine (10^−5^ mol/L). To investigate the NO-related pathways in response to acetylcholine, vasoreactivity was also evaluated after 45 min incubation with the NO synthase inhibitor L-NG-nitro-arginine (L-NNA, 10^−4^ mol/L).

### 4.4. Glycocalyx Analysis

Glycocalyx degradation was measured using enzyme-linked immunosorbent assay kits (ELISA) for heparan sulfate and syndecan-1 in plasma (Rat Syndecan-1 and Rat heparan sulfate, Quantikine, R&DSystem, Minneapolis, MN, USA).

### 4.5. Transmission Electron Microscopy (TEM)

The left pulmonary artery was prepared as previously described [26]. Detailed procedures are described in Supplementary Material S2.

### 4.6. Systemic Inflammation

Systemic inflammation was measured using ELISA for IL-1β, IL-10 and TNF-α in plasma. Detailed procedures are described in Supplementary Material S3.

### 4.7. Histology and Immunostaining Studies

Fluorescent immuno-histological marking, specific for macrophages (CD68) and T lymphocytes (CD3). Detailed methodologies procedures are detailed in Supplementary material S3.

### 4.8. Clinical Parameters

Heart rate and invasive blood pressure were monitored continuously during the procedure with the PowerLab^®^ device (AD Instruments, Sydney, Australia). Pulmonary oedema was measured using dry weights after 5 days at 65 °C. MAP was maintained between 50 and 60 mmHg during CPB using Gelofusine, HA, or HSL, depending on each group if necessary.

### 4.9. Statistical Analysis

All statistical analysis was performed with GraphPad Prism^®^8.2 (GraphPad Sofware Inc., San Diego, CA, USA). In the absence of data regarding the potential effect of CPB with HA or HSL prime on pulmonary vascular reactivity (primary outcome), we performed a simulation, using our historical data, of the effect of CPB with Gelofusine prime on pulmonary vascular reactivity to demonstrate statistical significance (*p* < 0.05) with a minimal power of 80% [5]. The minimal expected effect on relaxation between IR-CPB groups was fixed at 40%. Thus, at least five rats per group were required. With mortality and different losses, we planned to include eight rats per group. For all significant differences concerning primary endpoints, a posteriori powers higher than 80% were also checked. The Gaussian distribution of data was assessed using the Kolmogorov–Smirnov test before applying parametric tests as a one-sample *t*-test or Analysis of Variance (ANOVA) followed by Tukey’s multiple comparison test. The Mann–Whitney test or Kruskal–Wallis test, followed by Dunn’s multiple comparison test, were used for non-parametric distribution. Results for vascular studies results are expressed as mean ± standard error of the mean (SEM), and the other results are expressed as mean ± standard deviation (SD).

For statistical analyses of the glycocalyx degradation, the systemic inflammation, the immunohistology, the lung-score injury, and the clinical parameters, ANOVA was used followed by Tukey’s multiple comparison (post hoc) testing. A result with *p* < 0.05 was considered to be statistically significant.

## 5. Conclusions

We have demonstrated that HA used as a prime in CPB significantly decreased the effects of pulmonary IR on pulmonary vascular dysfunction compared to Gelofusine. However, we did not find any difference concerning inflammation and our study does not allow us to conclude on the use of HSL. Studies using HA as priming during LTx with mechanical circulatory support could be interesting to perform in the future.

## Data Availability

The data underlying this article will be shared on reasonable request to the corresponding author.

## Supplementary Materials

The following supporting information can be downloaded at: https://www.mdpi.com/article/10.3390/ijms23168938/s1.
