# Supplementary material for: Priming of Cardiopulmonary Bypass with Human Albumin Decreases Endothelial Dysfunction after Pulmonary Ischemia–Reperfusion in an Animal Model
Source: Int J Mol Sci. 2022 Aug 11;23(16):8938. doi: 10.3390/ijms23168938 (PMC9408928; doi:10.3390/ijms23168938)
Supplement: Supplementary file 1 [file ijms-23-08938-s001.zip › Supplementary Figures.pdf]

Supplementary Legends for Supplementary Figure S1 and S2

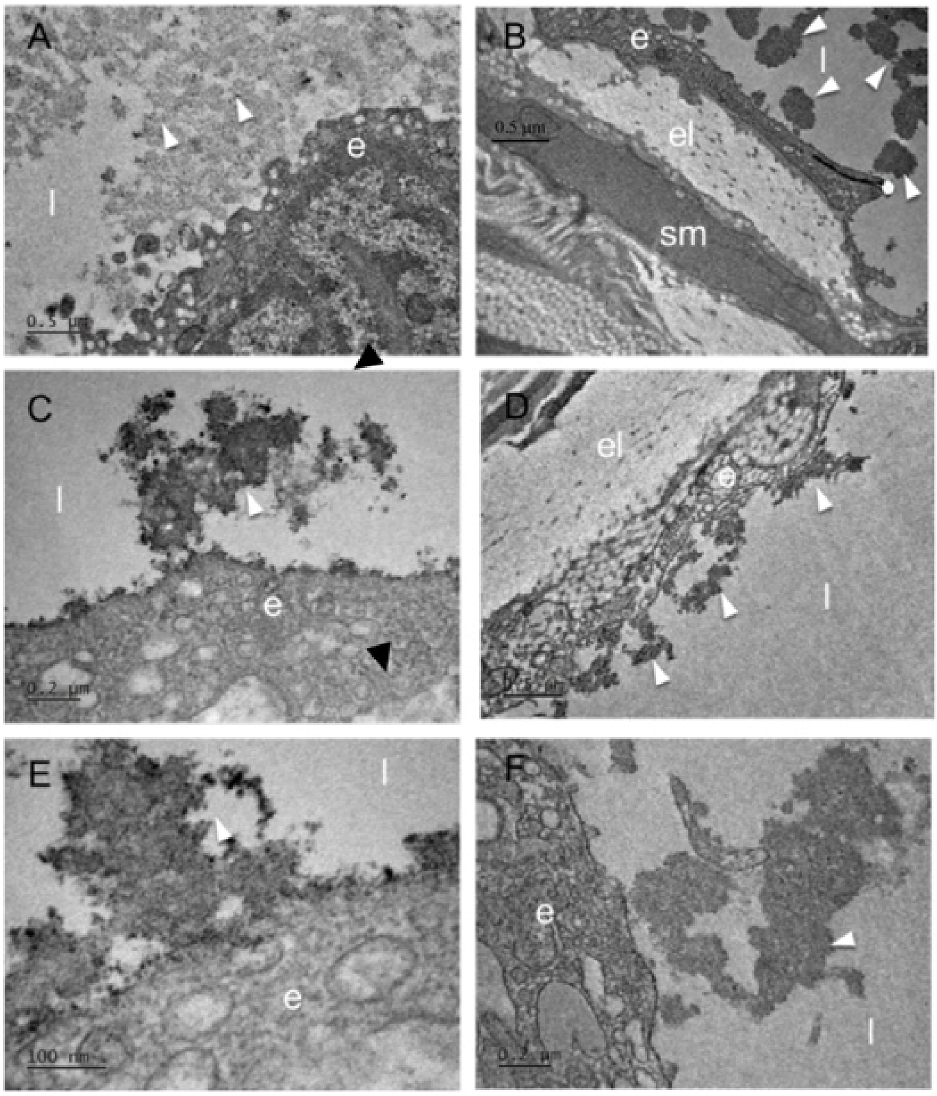

Supplementary Figure S1: Visualization of the luminal space of the pulmonary endothelial glycocalyx with transmission electronic microscopy. Large packed compounds (white head arrow) **(A)(B)(C)(D)(E)(F)**, and glycocalyx (black head arrow) **(C)(E)** are observed at the outer surface of the endothelium and in the lumen of artery. **(A)** IR-CPB-GF group, scale bar=0.5 µm. **(C)** IR-CPB-GF group, scale bar=0.2 µm. **(E)** IR-CPB-GF group, scale bar=100 nm **(B)** IR-CPB-HA group, scale bar=0.5 µm. **(D)** IR-CPB-HA group, scale bar=0.5 µm. **(E)** IR-CPB-HA group, scale bar=0.2 µm. Abbreviations: CPB, cardiopulmonary bypass; e, endothelium; el, elastic lamina; l, Lumen; IR, left lung ischaemia-reperfusion; HA, human albumin; sm, smooth muscle.

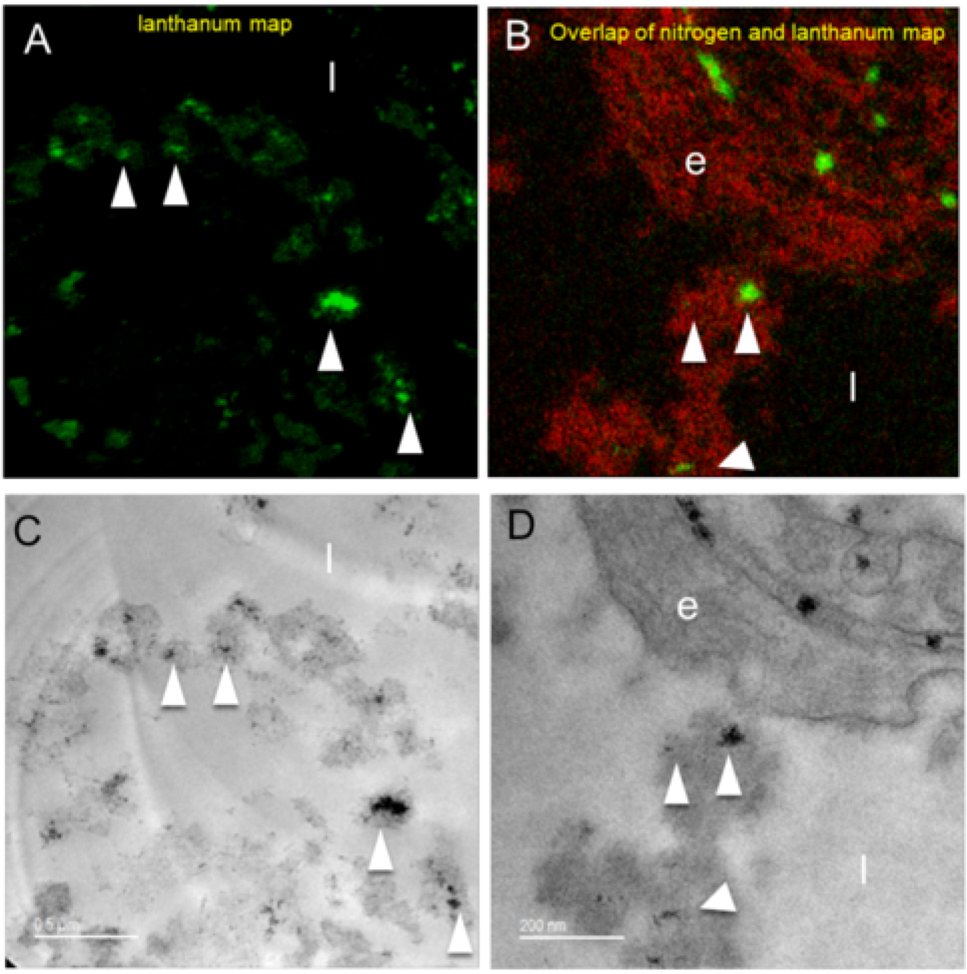

Supplementary Figure S2: Visualization of the interaction between glycocalyx and supposed prime solution in the lumen of the pulmonary artery by energy-filtered transmission electron microscopy (EFTEM). **(B)** Nitrogen map (red) by EFTEM confirmed the proteic nature of the luminal compound, supposed to be HA. Nitrate lanthanum staining showing the interaction between the glycocalyx (green spots by EFTEM **(A)(B)** or white head arrow with TEM **(C)(D)**) and the luminal compound, supposed to be HA (red). **(A)(C)** IR-CPB-GF group, scale bar=0.5 mm **(B)(D)** IR-CPB-HA group, scale bar=200 nm. Abbreviations: CPB, cardiopulmonary bypass; e, endothelium; I, Lumen; IR, left lung ischaemia-reperfusion; HA, human albumin.
