# Supplementary material for: Priming of Cardiopulmonary Bypass with Human Albumin Decreases Endothelial Dysfunction after Pulmonary Ischemia–Reperfusion in an Animal Model
Source: Int J Mol Sci. 2022 Aug 11;23(16):8938. doi: 10.3390/ijms23168938 (PMC9408928; doi:10.3390/ijms23168938)
Supplement: Supplementary file 1 [file ijms-23-08938-s001.zip › Supplementary material S1.pdf]

Supplementary material S1

Transmission electron microscopy (TEM)

To explore the impact of the prime solution on the glycocalyx, segments of pulmonary arteries were studied by TEM. TEM observations revealed that the lumen of the artery contains a high density of large packed compounds that may match to CPB prime solution (Supplementary Figure S1). A higher interaction of these compounds with the endothelium is observed in the IR-CPB-HA group compared to the IR-CPB-GF group (Supplementary Figure S1). The proteic nature of those compounds was confirmed by energy-filtered TEM micrographs (EFTEM) showing the nitrogen reactions. In addition, we observed a specific affinity between the lumen compounds and nitrate lanthanum used in TEM as a dense electron glycocalyx marker, suggesting an interaction between the HA and the glycocalyx (Supplementary Figure S2).
