# Supplementary material for: Priming of Cardiopulmonary Bypass with Human Albumin Decreases Endothelial Dysfunction after Pulmonary Ischemia–Reperfusion in an Animal Model
Source: Int J Mol Sci. 2022 Aug 11;23(16):8938. doi: 10.3390/ijms23168938 (PMC9408928; doi:10.3390/ijms23168938)
Supplement: Supplementary file 1 [file ijms-23-08938-s001.zip › Supplementary material S2.pdf]

Supplementary material S2

Transmission electron microscopy

Before sacrifice, rats were perfused through the left femoral vein with heparin, 2% glutaraldehyde, 2% sucrose supplemented with 2% nitrate lanthanum hexahydrate in cacodylate buffer 0.1 M pH 7.4. After excision, lungs were dissected out under stereomicroscope in order to isolate the pulmonary artery and obtain small cross-sections of pulmonary tissue (four per rat). During the dissection steps, tissues were kept immersed in a cold fixative solution to preserve them from collapse or degradation by air contact. Lung slices were fixed again by immersion for an additional 1 h in the same fixative solution used for perfusion. After a brief rinse in buffer, they were first, post-fixed 1 h at +4°C with 2% osmium tetroxide (EMS) then dehydrated with a gradual series of ethanol ending with dry acetone at 4°C. Infiltration was performed with Epon® resin (Polysciences, PA, USA) as a usual method. Ultrathin sections (50-70 nm, ultracut UCT, Leica, Nanterre, France)) were obtained on gold 600 mesh grids. The acquisitions were proceed on JEM-ARM200F HR-TEM. Elemental maps were acquired at 80kV in Energy Filtered -TEM mode (EFTEM) (GIF-Gatan-Ametek, Pleasanton, United States).

Surgical procedure

Rats were anesthetized with an intraperitoneal injection of xylazine (10 mg/kg) and ketamine (80 mg/kg). The left carotid was cannulated with a 22-gauge catheter (Introcan® Safety B. Braun) for continuous monitoring of heart rate, blood pressure (LabChart, ADInstruments®), and drug delivery. During the entire procedure, rectal temperature was continuously monitored. Heparin (500 IU/kg) was injected after conditioning and ketamine every 30 minutes. For mechanical ventilation (Ugo Basile®, Rodent ventilator), a tracheotomy

was performed with a 16-gauge catheter (Introcan® Safety B. Braun) with a respiratory frequency of 75 cycles/min, a tidal volume of 6 mL/kg and, inhalation of air during CPB and 100% O_2 during other situations. The right femoral artery and the right femoral vein were cannulated respectively by a 22-gauge catheter and a 16-gauge catheter (Introcan® Safety B. Braun). For CPB, we used a CPB sterile circuit including a roller pump (Fresenius Apparatebau), a cardiotomy reservoir consisting of a 5 mL syringe (Terumo®), an oxygenator (Kewei Medical Instrument Inc), and tubing lines. The oxygenator was connected to an oxygen tank. The circuit was aseptically set up and « free from air bubbles » with a 10 mL solution of 4% Gelofusine solution (4% Gelofusine®, B. Braun), 5% HA (5% Human Albumin, Vialebex®) or 11.2% HSL (11.2% sodium lactate (1000 mmol/L of sodium + 1000 mmol/L of lactate, APHP) according to the group. The arterial cannula was then connected to the right femoral artery and the venous cannula to the right femoral vein. The rat was positioned on a heated surface in a 30° proclive position to improve venous drainage. The CPB outflow was progressively increased to 100 mL/kg/min. The vascular filling was realized with Gelofusine or HA, or HSL depending of the group, allowing sufficient venous return for CPB, and additional infusion was performed in case of mean arterial pressure (MAP) inferior to 55 mmHg.

A thoracotomy was then performed in each group by a left intercostal incision under the axillary hollow. The left pulmonary hilum was carefully tightened with a lasso (Gore-Tex® Suture, Gore Medical) for ischaemia and released for reperfusion. Rats were exposed to 30 minutes of left pulmonary ischaemia (left hilum clamping) and 15 minutes of reperfusion. The rats were euthanized at the end of the study, for the sham group, rats were euthanized 45 minutes after conditioning. Blood samples were collected in all groups at two time points: T1) immediately after the surgical procedure and before CPB, T2) before the sacrifice.
