# Supplementary material for: Priming of Cardiopulmonary Bypass with Human Albumin Decreases Endothelial Dysfunction after Pulmonary Ischemia–Reperfusion in an Animal Model
Source: Int J Mol Sci. 2022 Aug 11;23(16):8938. doi: 10.3390/ijms23168938 (PMC9408928; doi:10.3390/ijms23168938)
Supplement: Supplementary file 1 [file ijms-23-08938-s001.zip › Supplementary material S3.pdf]

Supplementary File S3

Systemic inflammation

Blood samples at T1 and T2 were centrifuged 20 minutes at 2000 g, the plasma was recovered and immediately frozen at -80 °C until further analysis. Anti-inflammatory cytokines were measured using ELISA for IL-10 (Rat IL10 RL1000 Quantikine, R&DSystem) and proinflammatory plasma cytokines using ELISA for IL-1β (Rat IL-1β/IL-1F2 RLB00 Quantikine, R&DSystem), TNF-α (Rat TNF-α RTA00 Quantikine, R&DSystem) for the IR-CPB-GF group and IR-CPB-HA group. Kits were used in accordance with the manufacturer's guidelines, and analyses were read using a plate reader at an optical density of 450 nm.

Histology and immunostaining studies

For histologic studies, the left lung apex was removed and placed in Krebs solution, then in water-soluble glycols and resins (Tissue-Tek®, MicromMicrotech, Brignais, France) just before snap freezing and were then stored at -80°C. Lungs were cut into 7 µm-thick sections using a cryostat. All the sections were stained with fluorescent immuno-histological marking, specific for macrophages (CD68, dilution 1/200, BioRadMCA341R) and T lymphocytes (CD3, dilution 1/50, DAKO AO452) and examined by fluorescent microscopy. Five photographic images were taken per section and manual counting was performed with ImageJ® software (v1.50).
